# Novel Combination *BMP7* and *HGF* Gene Therapy Instigates Selective Myofibroblast Apoptosis and Reduces Corneal Haze In Vivo

**DOI:** 10.1167/iovs.17-23308

**Published:** 2018-02

**Authors:** Suneel Gupta, Michael K. Fink, Arkasubhra Ghosh, Ratnakar Tripathi, Prashant R. Sinha, Ajay Sharma, Nathan P. Hesemann, Shyam S. Chaurasia, Elizabeth A. Giuliano, Rajiv R. Mohan

**Affiliations:** 1 Harry S. Truman Memorial Veterans' Hospital, Columbia, Missouri, United States; 2 One-Health One-Medicine Ophthalmology and Vision Research Center, University of Missouri Columbia, Missouri, United States; 3 GROW Research Laboratory, Narayana Nethralaya Foundation, Bangalore, India; 4 Chapman University School of Pharmacy, Irvine, California, United States; 5 Mason Eye Institute, University of Missouri School of Medicine, Columbia, Missouri, United States

**Keywords:** *HGF*, *BMP7*, PEI-GNP, gene therapy, corneal fibrosis

## Abstract

**Purpose:**

We tested the potential of bone morphogenic protein 7 (*BMP7*) and hepatocyte growth factor (*HGF*) combination gene therapy to treat preformed corneal fibrosis using established rabbit in vivo and human in vitro models.

**Methods:**

Eighteen New Zealand White rabbits were used. Corneal fibrosis was produced by alkali injury. Twenty-four hours after scar formation, cornea received topically either balanced salt solution (BSS; *n* = 6), polyethylenimine-conjugated gold nanoparticle (PEI2-GNP)-naked plasmid (*n* = 6) or PEI2-GNP plasmids expressing *BMP7* and *HGF* genes (*n* = 6). Donor human corneas were used to obtain primary human corneal fibroblasts and myofibroblasts for mechanistic studies. Gene therapy effects on corneal fibrosis and ocular safety were evaluated by slit-lamp microscope, stereo microscopes, quantitative real-time PCR, immunofluorescence, TUNEL, modified MacDonald-Shadduck scoring system, and Draize tests.

**Results:**

PEI2-GNP–mediated *BMP7*+*HGF* gene therapy significantly decreased corneal fibrosis in live rabbits in vivo (Fantes scale was 0.6 in *BMP7*+*HGF*-treated eyes compared to 3.3 in −therapy group; *P* < 0.001). Corneas that received *BMP7*+*HGF* demonstrated significantly reduced mRNA levels of profibrotic genes: *α-SMA* (3.2-fold; *P* < 0.01), fibronectin (2.3-fold, *P* < 0.01), collagen I (2.1-fold, *P* < 0.01), collagen III (1.6-fold, *P* < 0.01), and collagen IV (1.9-fold, *P* < 0.01) compared to the −therapy corneas. Furthermore, *BMP7*+*HGF*-treated corneas showed significantly fewer myofibroblasts compared to the −therapy controls (83%; *P* < 0.001). The PEI2-GNP introduced >10^4^ gene copies per microgram DNA of *BMP7* and *HGF* genes. The recombinant *HGF* rendered apoptosis in corneal myofibroblasts but not in fibroblasts. Localized topical *BMP7*+*HGF* therapy showed no ocular toxicity.

**Conclusions:**

Localized topical *BMP7*+*HGF* gene therapy treats corneal fibrosis and restores transparency in vivo mitigating excessive healing and rendering selective apoptosis in myofibroblasts.

Injuries and infections of the eye compromise corneal transparency, which accounts for two-thirds of the eye's refraction and results in vision loss in an estimated 1.3 million Americans annually. Worldwide, corneal disorders are the third leading cause of preventable blindness.^1,2^ Despite increasing knowledge about the molecular mechanisms underlying corneal scarring,^3^ currently available therapies^4^ rely primarily on steroids and other drugs that carry the risk of multiple side effects,^5^ require repeated applications, and are often ineffective in restoring vision completely. Although mitomycin C (MMC) is a commonly used topical treatment for corneal fibrosis,^6^ its use continues to be the subject of debate due to its long-term side effects.^7^ A limbal graft is the treatment of choice in countries where donor corneal limbus is available.^8^ Corneal transplantation remains the gold standard for the treatment of corneal scars and restoration of vision.^9^ In 2015 alone, 48,792 corneal transplantations were performed in the United States, according to the Eye Bank Association of America (http://restoresight.org/wp-content/uploads/2016/03/2015-Statistical-Report.pdf), and about 12.7 million people in the world are awaiting donor corneal tissues.^10^ Besides the limited availability of donor corneas, a high immunologic rejection rate is another limiting factor for restoring vision from corneal transplantation. Thus, there is an undisputable need for the development of effective and safe nonsurgical targeted treatments for corneal fibrosis, including gene-based therapies that would provide long-term effectiveness, require minimal clinical follow up, and show minimal side effects.

Studies show that injury and infection to the cornea/eye leads to the activation of quiescent stromal keratocytes in the cornea, which then migrate to the wound site and transdifferentiate to a wound-repair phenotype, referred to as myofibroblasts.^4^ While molecular signaling and secretory functions of myofibroblasts are essential for proper corneal wound repair, their continued production and prolonged presence in the stroma lead to corneal fibrosis (haze or scarring) due to the extended activity of TGF-β signaling during corneal wound repair.^11^ The activation and differentiation of keratocytes have been shown to occur as a response to *IL-1α*/*IL-1β*, which are released by corneal epithelial cells after injury.^12^ In addition, multiple molecules, factors, ligands, and cytokines, including TGF-β released from injured epithelial cells, play a pivotal role in the induction of inflammation and corneal scarring due to excessive biological activities and fusion of extracellular matrix (ECM) and cytoskeletal proteins.^3,4^ TGF-β signaling is largely responsible for the transdifferentiation of keratocytes into myofibroblasts. The persistence of myofibroblasts after wound healing is known to be a major factor in the pathogenesis of corneal fibrosis and opacity, and ultimately, vision impairment.^3,13^

The TGF-β superfamily proteins activate downstream signaling via the Smad family of proteins.^14^ Bone morphogenic protein (BMP) belongs to the TGF-β superfamily and plays a significant role in ECM synthesis, tissue repair, and remodeling processes during corneal wound healing.^15^ The signaling protein BMP7 was originally described to have a significant role in the development of mammalian organs such as the kidney and the eye.^16^ BMP7 binds to the type I and II receptors and regulates receptor-regulated Smads (Smad1, Smad5, and Smad8) and inhibitory Smads (Smad6 and Smad7) in a complex wound-healing signaling network.^17^ In addition to BMP7, the expression of hepatocyte growth factor (HGF) and its receptor proteins has been found in the cornea, lacrimal glands, and tears.^18,19^ HGF has been identified as a mitogen that functions through the c-Met receptor tyrosine kinase in the protection and regeneration of organs^20^ as well as in the modulation of corneal repair.^21,22^ Injury to corneal epithelium has been shown to upregulate HGF expression in keratocytes, and in addition to its intracrine and autocrine functions, HGF has been shown to function in a paracrine manner in modulating corneal wound healing.^23^ The role of HGF and the c-Met system in diabetic corneal wound healing was recently well established in organotypic human diabetic corneal cultures.^4^ Furthermore, HGF has been reported to have a role in the breakdown of ECM deposits and in the reduction of fibrosis in several nonocular tissues.^24^ Despite the important role of HGF in corneal wound healing and TGF-β profibrotic signaling, mechanistic knowledge about the crosstalk between HGF and BMP7 in the cornea remains unknown, especially during wound healing and profibrotic microenvironment.

The cornea represents a perfect tissue for gene therapy because of its well-defined characteristics such as transparency, simple anatomy, and ease of access, allowing topical instillation of gene delivery vectors and visual monitoring of the genes packaged within vectors.^25^ In addition, therapeutic response can also be assessed noninvasively with high-resolution ocular imaging using stereo and slit-lamp biomicroscopy.^26^ Our group previously reported the advantages of tissue-specific gene delivery using a variety of modalities, including direct instillation of hybrid nanoparticle or adeno-associated virus vectors.^17,27,28^ We found that nonviral gene delivery systems based on synthetic polycations,^29–31^ such as polyethylene amine (PEI), show promise as delivery systems for gene therapy. They possess DNA-binding capabilities, provide options for functionalization, and show a good safety profile. In subsequent studies, we greatly enhanced the otherwise low transfection efficiency of PEI (2 kDa) by conjugating PEI with gold nanoparticles to synthesize PEI-conjugated gold nanoparticles (PEI2-GNP).^32^ Utilizing these hybrid gold nanoparticles, we established a novel nanoparticle-based gene delivery system for the cornea that demonstrates efficient gene transfer into rabbit corneal stroma in vivo with negligible toxicity.^17,29^

BMP7 and HGF are attractive targets for modulating the profibrotic signaling pathways in corneal wound healing. Our previous study of *BMP7* gene therapy by PEI2-GNP in the preclinical rabbit model of corneal fibrosis revealed that PEI2-GNP–based *BMP7* gene therapy led to significant inhibition of corneal fibrosis and corneal repair through counterbalancing the deleterious effects of TGF-β–induced Smad signaling.^17^ *HGF* gene therapy has been shown by other investigators to regulate fibrosis in various nonocular tissues, including the liver,^20^ lung,^33^ and kidney.^34^ Phase-I and phase-II clinical trials indicate *HGF* gene therapy is safe in humans.^35,36^ Furthermore, *HGF* gene transfer has been found to cause selective apoptosis of myofibroblasts in nonocular tissues.^37–39^ These reports led to an innovative postulate that PEI2-GNP–mediated tissue-targeted localized *BMP7*+*HGF* gene therapy in rabbit cornea would effectively eliminate preexisting fibrosis in vivo without producing significant toxicity. The present study tested the therapeutic potential of PEI2-GNP–delivered *BMP7*+*HGF* gene therapy for abolishing preexisting corneal fibrosis in vivo using a well-established preclinical rabbit model of corneal fibrosis.

## Materials and Methods

### Corneal Fibrosis Induction and Treatment in Rabbits

Eighteen New Zealand White female rabbits, weighing 2 to 3 kg (Covance Research Products, Denver, PA, USA) were used in the study. Institutional approval of the study was obtained from the Harry S. Truman Memorial Veterans' Hospital and the Institutional Animal Care and Use Committee of the University of Missouri (both in Columbia, MO, USA). All animals were treated in accordance with the principles of the ARVO Statement for the Use of Animals in Ophthalmic and Vision Research. The rabbits were anesthetized by a mixture of ketamine hydrochloride (50 mg/kg) and xylazine hydrochloride (10 mg/kg), given intramuscularly, for induction of corneal alkali-induced wounding, for administration of PEI2-GNP–mediated *BMP7*+*HGF* gene delivery to the corneal stroma, and for the performance of clinical slit-lamp eye examinations and ocular stereo biomicroscopy. Topical ophthalmic proparacaine hydrochloride (0.5%; Alcon, Fort Worth, TX, USA) was administered for local anesthesia prior to all procedures.

### IOP Monitoring by Tonometry

Variations in IOP, an indicator of an ocular abnormality, may result from inflammation, swelling, rigidity, abrasion, and irregularities in corneal tissues. Administration of therapeutic genes into stroma has potential for alterations in the aqueous humor or in tissues of the anterior chamber, which is a significant concern after gene therapy. Thus, IOP measurements in rabbit eyes were recorded using a tonometer (Tono-Pen AVIA; Reichert Technologies, Depew, NY, USA) at regular timed intervals on days 1, 7, 14, and 21 and before each clinical biomicroscopy evaluation as reported earlier.^40^ All IOP measurements were performed between 9 AM and 11 AM to minimize normal diurnal variations in IOP.

### In Vivo Alkali-Induced Corneal Scarring

Corneal scarring was induced in one eye of each rabbit, and the contralateral eye served as a naive control. To induce corneal scarring, rabbits were anesthetized and an 8-mm filter paper soaked in 0.5 N sodium hydroxide solution was applied onto the central cornea for 1.0 minutes under visualization with the surgical microscope (Leica Wild Microscope MEL53; Leica, Wetzlar, Germany). The wounded corneas were immediately and copiously rinsed with sterile balanced salt solution (BSS) to remove alkali residual. This method triggered wound healing and produced dense corneal scarring and peak fibrosis at 3 weeks with minimal neovascularization.^41^

### PEI2-GNP Transfection Solution

Thiol-modified PEI2-GNPs were synthesized as described earlier.^32^ The PEI2-GNP transfection solution was prepared as reported previously.^17^ In brief, the PEI2-GNPs were mixed with plasmid at a nitrogen-to-phosphate (N/P) ratio of 180 by stirring 37.5 μL of 150 mM PEI2-GNPs with 10 μg plasmid DNA (pTRUF11 expressing *HGF* or *BMP7* under control of hybrid cytomegalovirus [CMV] chicken β-actin promoter), 10% glucose (wt/vol), and bringing the volume to 100 μL with BSS. The solution was incubated at 37°C for 30 minutes prior to application on the cornea.

### In Vivo Gene Delivery

One eye of each animal was treated and the contralateral eye served as naive control. To determine the effectiveness of gene therapy for preexisting corneal fibrosis, PEI2-GNP–mediated *BMP7*+*HGF* gene therapy was delivered into rabbit stroma 24 hours after alkali injury. BSS or transfection solution was topically applied to the cornea for 5 minutes using a cloning cylinder, as previously reported.^17,29^ The rabbits were divided into three groups: group 1 rabbits received BSS alone (*n* = 6; no gene transfer naive group); group 2 rabbits received PEI2-GNP-naked plasmid without *BMP7* or *HGF* gene (*n* = 6; −therapy group); and group 3 rabbits (*n* = 6) received PEI2-GNP plasmids expressing *HGF* and *BMP7* genes (*n* = 6; +therapy group). The cloning cylinder method is known to deliver significant levels of therapeutic genes into rabbit stroma in vivo with low toxicity.^17,29^

### Slit-Lamp Biomicroscopy, Haze Quantification, and Fluorescein Eye Test

Corneal defects and general ocular health were documented at baseline and after alkali wounding at various time points using a handheld slit-lamp microscope equipped with a digital-imaging system (SL-15; Kowa Optimed, Torrance, CA, USA). Using the Fantes scale, the intensity of corneal haze was graded by three independent observers (SG, AS, MK) masked to the treatment group, as reported earlier.^28,42^ In brief, the grading system was the following: grade 0, completely clear cornea; grade 0.5, trace haze seen with careful oblique illumination; grade 1, more obvious haze but not interfering the visualization of fine iris details; grade 2, mild obscuration of iris details; grade 3, moderate obscuration of the iris and lens; and grade 4, complete opacification of the stroma in the area of ablation.

Fluorescein sodium sterile ophthalmic strips (0.6 mg, Ful-Glo; Akorn, Lake Forest, IL, USA) were used to evaluate the health of the corneal epithelium and tear production. In brief, a BSS-moistened fluorescein strip was applied to the dorsal upper eyelid of rabbits, and the rabbits were allowed to blink for distributing fluorescein stain over the entire corneal and conjunctival surface. After 30 seconds, corneal epithelium was observed under blue light using wide and narrow beams of the slit-lamp microscope. Images were obtained with a stereo microscope fitted with a fluorescence filter, spot camera, and imaging software. Tear levels were measured using commercial diagnostic strips (Tear-Flow Diagnostic Strips; HUB Pharmaceuticals, Rancho Cucamonga, CA, USA).

### Ocular Irritation Tests

The ocular health and anomalies were evaluated independently at selected times by at least two of three examiners (MF, AS, and SG) using the established Draize^43^ and modified MacDonald-Shadduck^44^ ocular scoring systems. With the Draize eye test, the severity of ocular lesions was scored in the following manner: in the cornea, by estimating the degree of opacity and area of involvement; in the iris, by examining pupillary light reflexes; and in the conjunctiva, by assessing the degree of redness, chemosis, and discharge. With the modified MacDonald-Shadduck scoring system, ocular health scores were determined based on the cumulative average scores for corneal tissue (opacity, affected area, corneal neovascularization severity, and reepithelization) and for conjunctival tissue (congestion, chemosis, swelling, and discharge).

### Euthanasia and Tissue Collection

Rabbits were humanely euthanized with pentobarbital (150 mg/kg) while they were under general anesthesia. Corneas were harvested, immediately placed in 15 × 5 × 5-mm molds (Fisher Scientific, Pittsburgh, PA, USA) containing optical cutting temperature (OCT) compound, and snap frozen by immersion in a cryo-cup containing 2-methylbutane sitting in liquid nitrogen. Frozen tissue blocks were preserved at −80°C. The corneas were cut in two equal halves; one half was used for histologic studies, and other half was used for molecular studies. For histologic studies, serial corneal sections (8 μm) were prepared with a cryostat (HM525 NX UV; Microm GmbH, Walldorf, Germany), placed on glass microscopic slides (Superfrost Plus; Fisher Scientific, Pittsburgh, PA, USA), and kept at −80°C until analysis. For molecular studies, corneas were cut into small pieces, immediately immersed in a cryo-cup placed in liquid nitrogen, and subsequently ground and processed for obtaining genomic DNA, mRNA, and cDNA following vendor's protocols (Qiagen, Germantown, MD, USA).

### Hematoxylin and Eosin, Masson's Trichome, Immunofluorescence and TUNEL Staining

Hematoxylin-eosin (H&E) and Masson's trichome staining were performed using the standard procedure for visualizing morphologic details, as reported earlier.^40,45^ Immunofluorescence staining was performed following reported methods^17^ to measure myofibroblasts in the corneas using antibody-specific α-smooth muscle actin (*α-SMA*), a marker for myofibroblasts. Briefly, corneal sections were blocked with 2% bovine serum albumin at room temperature for 30 minutes, followed by incubation with *α-SMA* mouse monoclonal primary antibody (1:200 dilution, M0851; Dako, Carpentaria, CA, USA) for 90 minutes and then incubated with Alexa-Fluor 488 goat anti-mouse IgG secondary antibody (1:1000 dilution, A11001; Invitrogen, Carlsbad, CA, USA) for 1 hour at room temperature. Appropriate positive and negative controls were included in each immunostaining. Quantification of *α-SMA*-positive cells was performed in six randomly selected, nonoverlapping, full-thickness central corneal columns, extending from the anterior stromal surface to the posterior stromal surface at 200× and 400× magnification fields.

The toxicity of PEI2-GNPs and *BMP7*+*HGF* gene therapy was determined by performing a TUNEL assay (ApopTag; Millipore, Temecula, CA, USA). Corneal sections were fixed in acetone at –20°C for 10 minutes, and a TUNEL assay was performed per the manufacturer's instructions, including suitable positive and negative controls. Rhodamine-conjugated apoptotic cells (red) and 4′,6-diamidine-2′-phenylindole dihydrochloride (DAPI)–stained nuclei (blue) were viewed and photographed with a fluorescence microscope (Leica) fitted with a digital camera system (SpotCamRT KE; Diagnostic Instruments, Sterling Heights, MI, USA). DAPI-stained nuclei and TUNEL-positive cells in untreated and treated tissues were quantified at 200× and 400× magnification in six randomly selected nonoverlapping areas, as previously reported.^17,28^

### RNA Extraction, cDNA Synthesis, and Quantitative Real-Time PCR

Total RNA from tissues was extracted with an RNeasy kit (Qiagen, Valencia, CA, USA) and reverse transcribed to cDNA following reported methods.^17,28^ Real-time PCR was performed using the StepOne Plus PCR system (Applied Biosystems, Carlsbad, CA, USA). A 20-μL reaction mixture contained 2 μL cDNA, 2 μL forward and reverse primers (200 nM each), and 10 μL 2X All-in-One quantitative PCR (qPCR) mix (GeneCopoeia, Rockville, MD, USA) and was run at a universal cycle (95°C for 10 minutes, 40 cycles at 95°C for 15 seconds, and 60°C for 60 seconds), as previously reported.^16^ The primer sequences of genes were the following: *α-SMA*—forward TGG GTG ACG AAG CAC AGA GC and reverse CTT CAG GGG CAA CAC GAA GC; fibronectin—forward CGC AGC TTC GAG ATC GTG C and reverse TCG ACG GGA TCA CAC TTC CA; collagen type I—forward TGT GGC CCA GAA CTG GTA CAT and reverse ACT GGA ATC CAT CGG TCA TGC TCT; collagen type III—forward AGA ACA CGC AAG GCT GTG AGA CTA and reverse CCA ACG TCC GCA CCA AAT TCT TGA; collagen type IV—forward TAT CGA ACA ACG CAA GGC TGT GAG A and reverse GGC CAA CGT CCA CAC CAA ATT CTT; and β-actin—forward CGG CTA CAG CTT CAC CA and reverse CGG GCA GCT CGT AGC TCT TC. The β-actin was used for the normalization of qPCR data, and showed no appreciable relative fold change at various tested points and groups.

### Quantification of Nanoparticle-Delivered Gene Copies

The frozen corneal tissues were pulverized in liquid nitrogen, and genomic DNA was isolated (DNA Easy kit; Qiagen, Valencia, CA, USA). Quantitative PCR was performed to determine PEI2-GNP–delivered gene copies of *HGF* and *BMP7* in corneal tissues.^17^ A 10-fold serial dilution of plasmid having test gene (10^4^–10^9^/mg DNA) was used for standard curves. The qPCR settings were 95°C for 10 minutes, 40 cycles at 95°C for 15 seconds, and 60°C for 60 seconds.

### Human Corneal Fibroblast and Myofibroblast Cultures

Primary human corneal fibroblast (HCF) cultures were generated from donor human corneas purchased from an eye bank (Saving Sight, Kansas City, MO, USA) following methods described previously.^30^ The corneal epithelium and endothelium were removed from corneal buttons with a surgical blade and cut into small pieces, placed on culture dishes, and incubated for 3 to 5 weeks in a humidified CO_2_ (5%) incubator at 37°C in MEM supplemented with 10% fetal bovine serum. Seventy percent confluent HCF cultures that underwent fewer than four passages were used in the experiments. Human corneal myofibroblasts were produced by culturing HCFs under serum-free conditions in the presence of TGF-β1 (PeproTech, Rocky Hill, NJ, USA), 5 ng/mL for 72 hours.

### Lactate Dehydrogenase Assay

Lactate dehydrogenase (LDH) assay was performed to measure cytoplasm enzyme LDH released in the extracellular medium. Briefly, 4.0 × 10^3^ cells were seeded in 96-well plates. LDH titers of fibroblast and myofibroblast cultures were assessed in the presence and absence of *HGF* conditions. After 24 hours, the supernatants were collected from each well. Cell monolayer was then treated with a cell lysis solution for 30 minutes at room temperature. Cell lysate and supernatant were collected. LDH activity in samples was measured using a toxicology assay kit (TOX7; Sigma-Aldrich Corp., St. Louis, MO, USA) following the manufacturer's instructions. The absorbance was determined at 490 nm, and LDH activity was expressed in international units per milliliter.

### Statistical Analysis

Quantification studies were performed using Student's *t*-test, 1-way and 2-way ANOVA followed by Bonferroni multiple comparisons test, or the Wilcoxon rank sum test and/or Tukey's multiple comparison tests. The results are expressed as mean ± standard error of the mean (SEM). *P* values < 0.05 are considered to be statistically significant.

## Results

### Effects of *BMP7*+*HGF* on Corneal Fibrosis and Corneal Transparency

**Figure 1:**
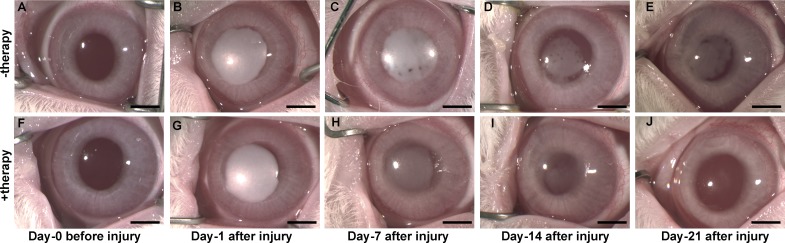
Biomicroscopy images before and after alkali-induced corneal injury of rabbit eyes that did not receive BMP7+HGF treatment (A–E) and those that received BMP7+HGF treatment (F–J). Dense corneal opacity developed by day 1 after induction of alkali injury (B, G). On day 14 and day 21 post injury, corneas that received PEI2-GNP–mediated BMP7+HGF eyes showed a significant reduction of opacification (H–J) as compared to the level of opacification in corneas that received naked vector (C–E). Scale bar: 2 mm.

PEI2-GNP–mediated *BMP7* and *HGF* gene therapy targeted to the corneal stroma significantly dissipated preexisting corneal fibrosis and restored corneal transparency in vivo (Figs. 1A–J). The corneas of all rabbits were transparent before injury (Figs. 1A, 1F) and developed dense scar/fibrosis 24 hours after alkali wound (Figs. 1B, 1G), and gene transfer was administered at this time. Rabbit corneas that received *BMP7*+*HGF* genes (+therapy group) not only demonstrated a marked reduction in corneal fibrosis but also concurrent increased transparency on day 7 (Fig. 1H), day 14 (Fig. 1I), and day 21 (Fig. 1J) as compared to the corneas that received naked vector (−therapy group; Figs. 1C, 1D, 1E).

**Figure 2:**
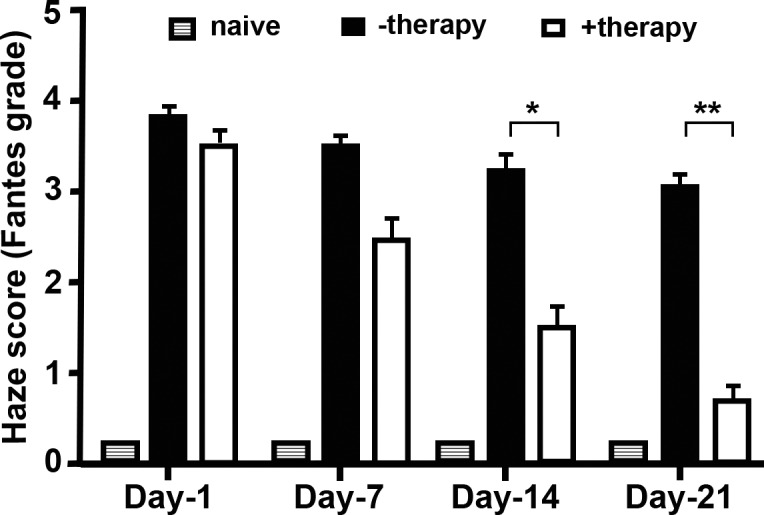
Quantification of corneal opacity using the Fantes score in the three groups of rabbits: no corneal injury group (naive; gray bar), PEI2-GNP-naked plasmid group (−therapy; black bar), and PEI2-GNP–BMP7+HGF group (+therapy; white bar). The graph depicts comparative haze scores 24 hours post injury and at days 7, 14, and 21 post injury (*P < 0.01 and **P < 0.001).

Figure 2 depicts quantitative corneal clinical haze scores, based on Fantes scoring, assigned by three independent observers (SG, MK, AS) masked to the treatment group after alkali wound at days 1, 7, 14, and 21. On days 14 and 21, corneas that received PEI2-GNP–mediated *BMP7*+*HGF* therapy showed markedly lower corneal haze scores as compared to BSS-treated corneas. The *BMP7*+*HGF* gene delivery rendered a statistically significant decrease in corneal fibrosis at day 14 (2.3-fold; *P* < 0.01) and day 21 (5.5-fold; *P* < 0.001) as compared to the corresponding naked vector–delivered control corneas. As expected, no significant difference in corneal haze scores was observed in the +therapy and −therapy groups on day 1 and day 7. The comparison of the +therapy versus naive eyes at day 1, 7, and 14 showed significant corneal haze, but not at day 21 (*P* = 0.215).

### Effects of *BMP7*+*HGF* on Healing of the Corneal Stroma and Epithelium

**Figure 3:**
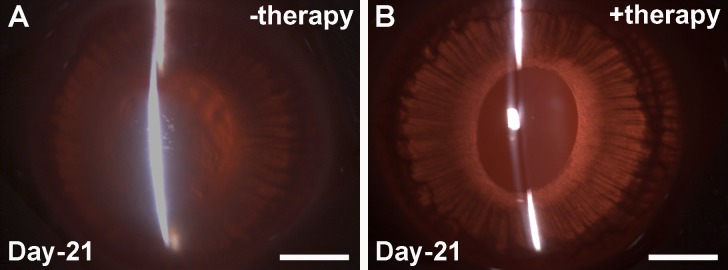
Slit-lamp images at day 21 after administration of PEI2-GNP only (A) and of PEI2-GNP–mediated BMP7+HGF (B), showing persistence of haze in the −therapy eye and a clear cornea after BMP7+HGF. Scale bar: 2 mm.

*BMP7*+*HGF* overexpression prevented excessive scarring in corneal stroma, and normal physiological healing was observed. Figures 3A and 3B show representative slit-lamp biomicroscopy images on day 21 in the −therapy and +therapy groups. A significant reduction in corneal haze and fibrosis was observed at day 21 in corneas that received PEI2-GNP–mediated *BMP7*+*HGF* (Fig. 3B) as compared to corneas that received PEI2-GNP-naked plasmid (Fig. 3A). Haze scores were 0.6 versus 3.3 (*P* < 0.001) in the +therapy and −therapy groups, respectively. Moreover, at day 21 subjective ocular examination of rabbit eyes in the *BMP7*+*HGF* group appeared normal in contrast to the eyes of the vector-only group, which showed mild edema, inflammation, redness, and discharge.

**Figure 4:**
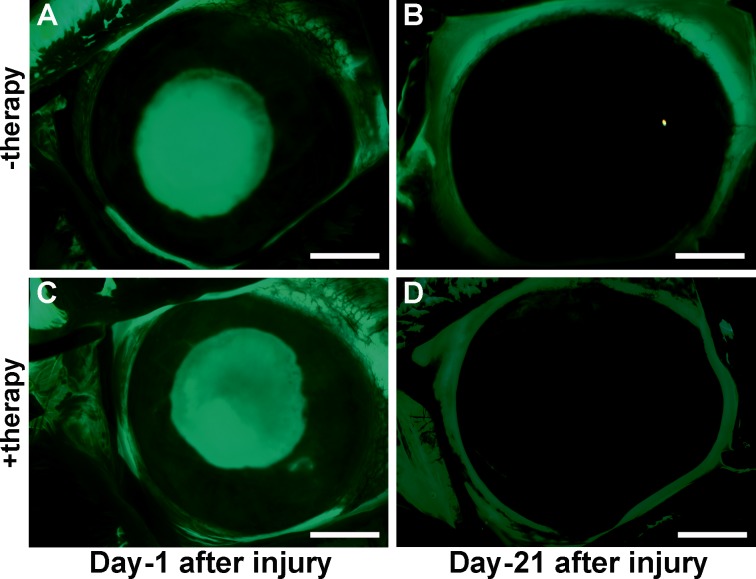
Stereo fluorescence microscopy images on days 1 and 21 post injury of eyes treated with PEI2-GNP alone (A, B) or BMP7+HGF (C, D), showing that therapy does not affect reepithelialization of the injured corneas. Scale bar: 2 mm.

Fluorescein staining revealed that *BMP7*+*HGF* gene transfer is not detrimental to re-epithelization of the injured corneas (Fig. 4). Fluorescence stereo biomicroscopy images showed corneal ulceration and epithelial defect on day 1 after alkali injury (Figs. 4A, 4C), but by day 21 all of the treated rabbits demonstrated complete corneal re-epithelialization and negative fluorescein staining (Figs. 4B, 4D). The lacrimal lake, as observed on tear strips, appeared similar in both PEI2-GNP-naked vector and *BMP7*+*HGF* groups, suggesting that combination therapy with *BMP7*+*HGF* does not compromise tear production or invoke a dry eye condition.

### Myofibroblast and Profibrotic Gene Expression

**Figure 5:**
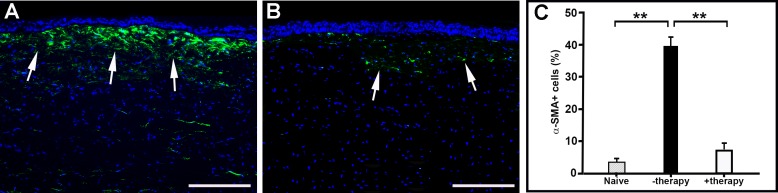
Immunofluorescence images showing stromal expression of α-SMA, a myofibroblast marker, in corneas 21 days post injury in rabbits that did not receive gene therapy (A) and those that received BMP7+HGF gene therapy (B). Quantification graph depicts the significant reduction of α-SMA in the +therapy group compared to the −therapy group (P < 0.001). Scale bar: 100 μm.

Determination of myofibroblast after alkali-induced corneal injury indicates that *BMP7*+*HGF* gene therapy has an inhibitory effect on myofibroblast formation and profibrotic gene expression (Fig. 5). Immunohistologic staining of *α-SMA*, a marker for myofibroblasts, demonstrated a clinically relevant and statistically significant reduction in *α-SMA*-positive cells in corneas that received *BMP7*+*HGF* genes (Fig. 5B) as compared to corneas of the naive and PEI2-GNP-naked plasmids (Fig. 5A). The immunofluorescence quantification graph (Fig. 5C) showed an 83% reduction of *α-SMA*-positive cells (*P* < 0.001) in the +therapy group as compared to the number of *α-SMA*-positive cells in the −therapy group (Fig. 5C).

**Figure 6:**
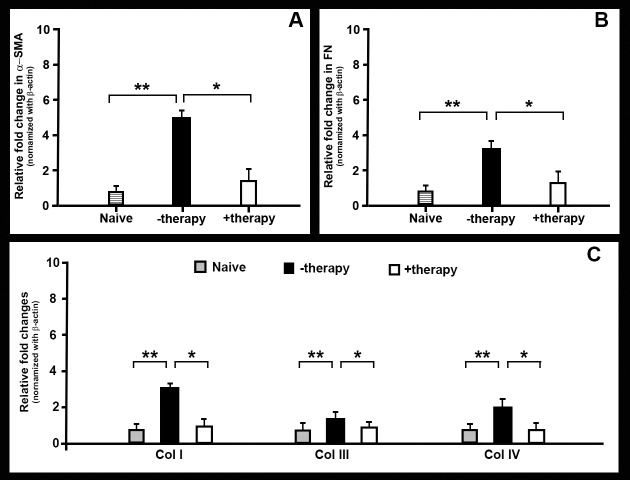
Bar graphs showing differential mRNA expression of (A) α-SMA, (B) fibronectin (FN), and (C) collagen I (Col I), collagen III (Col III), and collagen IV (Col IV) in the corneal tissue from rabbits in the naive, PEI2-GNP (−therapy), and BMP7+HGF (+therapy) groups. Tissue was obtained 21 days after injury. BMP7+HGF treatment reduced fibrotic gene expression in injured cornea. Quantification of mRNA expression of fibrotic-related genes by real-time PCR. Graphs depict relative fold expression of α-SMA, fibronectin, collagen I, collagen III, and collagen IV (n = 6 for each group). Error bars represent ± SEM (**P < 0.001 against naive control and *P < 0.01 against BMP7+HGF group, respectively).

Since *BMP7*+*HGF* gene delivery into fibrotic rabbit corneas led to a quantifiable improvement in wound healing after alkali burn in our in vivo model, the expression of five prominent genes (*α-SMA*, fibronectin, collagen I, collagen III, and collagen IV) involved in fibrosis pathways was also examined in the corneas of rabbits that received BSS, PEI2-GNP-naked plasmid or *BMP7*+*HGF* genes (Fig. 6). As Figure 6 illustrates, alkali injury significantly increased levels of the five tested profibrotic genes in the −therapy corneas compared to naive corneas from 2.2- to 5.2-fold (*P* < 0.001), whereas *BMP7*+*HGF* gene transfer significantly reduced profibrotic gene expression of the *α-SMA*, a 3.2-fold reduction (Fig. 6A; *P* <0.01); fibronectin, a 2.3-fold reduction (Fig. 6B; *P* <0.01); collagen I, a 2.1-fold reduction (Fig. 6C; *P* <0.01); collagen III, a 1.6-fold reduction (Fig. 6C; *P* <0.01), and collagen IV, a 1.9-fold reduction (Fig. 6C; *P* <0.01).

### *BMP7*+*HGF* Dissipates Myofibroblasts via Apoptosis

**Figure 7:**
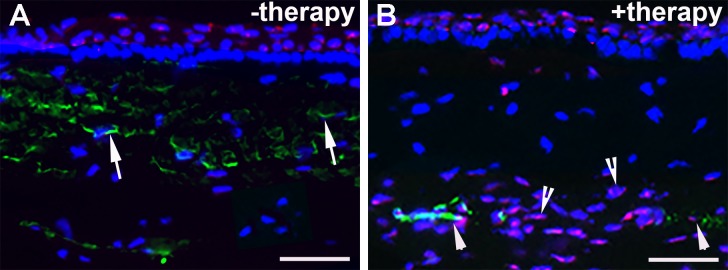
Double immunofluorescence staining for α-SMA and TUNEL in tissue from injured corneas treated with (A) PEI2-GNP alone (−therapy) and (B) PEI2-GN–delivered BMP7+HGF (+therapy). Arrows point to α-SMA; arrowheads point to colocalization of α-SMA-positive and TUNEL-positive cells; and arrowheads with inside tail point to TUNEL-positive cells. Scale bar: 25 μm.

Double immunofluorescence staining of corneal tissue sections for *α-SMA* (a myofibroblast marker) and TUNEL (an apoptosis marker) demonstrated that *HGF* delivery dissipates myofibroblasts through apoptosis in the fibrotic cornea. Figure 7 shows representative images of double immunofluorescence performed in corneal tissue collected on day 21 after injury. The anterior stromal tissue of *BMP7*+*HGF*-treated corneas was found to contain several double-stained *α-SMA*-positive and TUNEL-positive cells (4.7 ± 1.2/400×) and single-stained *α-SMA* cells (4.5 ± 1.1/400×) and TUNEL-positive cells (21.4 ± 5.7/400×) (Fig. 7B). In contrast, the anterior stromal tissue of corneas that received PEI2-GNP-naked vector showed only *α-SMA*-positive cells (41.7 ± 6.1/400×) (Fig. 7A). The difference between the +therapy and the −therapy group in the disappearance of myofibroblast via apoptosis was statistically significant (*P* < 0.001).

### Number of Gene Copies Driving Therapeutic Response

**Figure 8:**
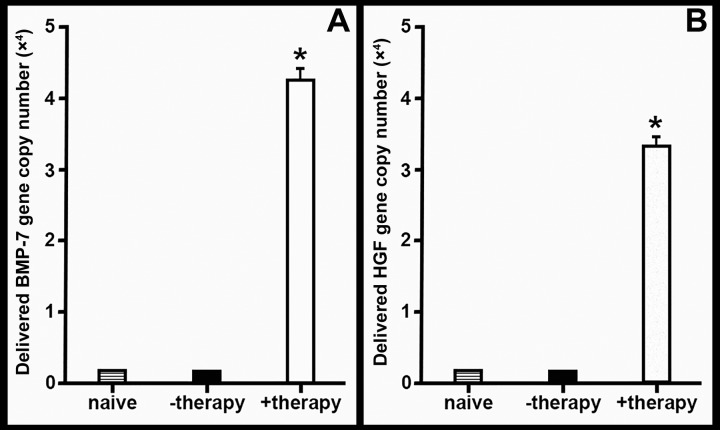
Bar graph of quantitative PCR analysis showing BMP7 (A) and HGF (B) gene copy numbers delivered by PEI2-GNPs to the rabbit cornea, indicating efficient delivery of genes by the customized nanoparticles. There were six samples in each group and error bars represent ± SEM (*P < 0.001 against naive control and −therapy group).

Real-time PCR quantification of the number of therapeutic gene copies responsible for reducing corneal fibrosis indicated that sufficient delivery of the genes was achieved with the PEI2-GNPs administered via cloning cylinder technique (Figs. 8A, 8B). Gene delivery by the PEI2-GNPs was significant, as 4.3 × 10^4^ ± 0.2 copies of *BMP7* per 1 μg DNA (Fig. 8A) and 3.2 × 10^4^ ± 0.4 copies of *HGF* per 1 μg DNA (Fig. 8B) were detected in treated corneas.

### In Vivo Toxicity and Safety Studies

The results of time-dependent ocular irritation studies performed in live rabbits using the Draize and modified MacDonald-Shadduck scoring systems are highlighted in the Table. As expected, an alkali wound led to a significantly increased cumulative Draize score in the PEI2-GNP-naked vector and PEI2–mediated *BMP7*+*HGF* group as compared to naive corneas. On day 7, the average Draize score was 45.0 in the −therapy group versus 0 in the naive group (*P* < 0.001); on day 14, 39.0 vs. 0 (*P* < 0.001); and on day 21, 29.8 vs. 0 (*P* < 0.001). *BMP7*+*HGF* therapy was associated with a significant, time-dependent reduction in the cumulative Draize score compared to the score in the −therapy group as follows: day 7, 29.1 with *BMP7*+*HGF* vs. 45.0 with −therapy (*P* < 0.01); day 14, 18.9 vs. 39.0 (*P* < 0.01); and day 21, 5.1 vs. 29.8 (*P* < 0.01). The modified MacDonald-Shadduck test results showed a similar pattern of scores following alkali wounding in PEI2-GNP-naked vector and *BMP7*+HFG groups. Injured eyes that received *BMP7*+*HGF* therapy exhibited significantly lower modified MacDonald-Shadduck scores than did the eyes of rabbits that received PEI2-GNP-naked vector (day 7: 0.9 vs. 1.8, *P* < 0.05; day 14: 0.6 vs. 1.3, *P* < 0.01; day 21: 0.3 vs. 1.1, *P* < 0.001). On the subjective clinical eye examinations performed independently by three examiners, significantly improved overall ocular health was observed in the eyes of rabbits that received gene therapy as compared to eyes of rabbits that received the nanoparticles alone.

**Table:**
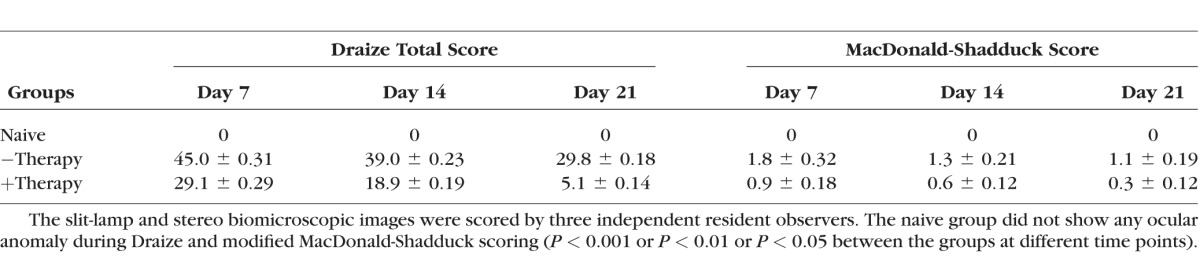
Draize and Modified MacDonald-Shadduck Scoring Shows the Ocular Anomaly in −Therapy and +Therapy Groups

**Figure 9:**
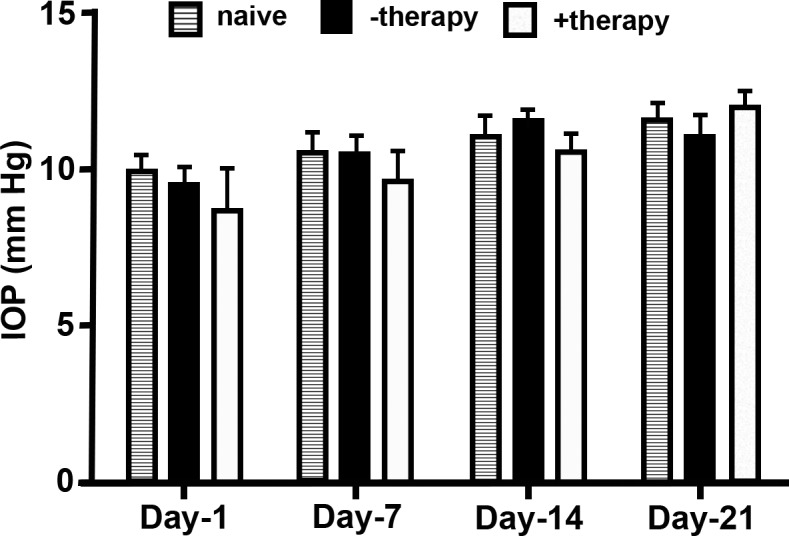
BMP7+HGF therapy is well tolerated in rabbit eyes. Graph depicts IOP of the animal groups under investigation. Error bars represent ± SEM and no significant difference (P > 0.1) was recorded between the groups.

Evaluation of the effects of *BMP7*+*HGF* therapy on IOP and tear production revealed no significant differences in the IOP (Fig. 9; *P* > 0.1) and tear levels (data not shown) in the eyes of rabbits among the three groups.

**Figure 10:**
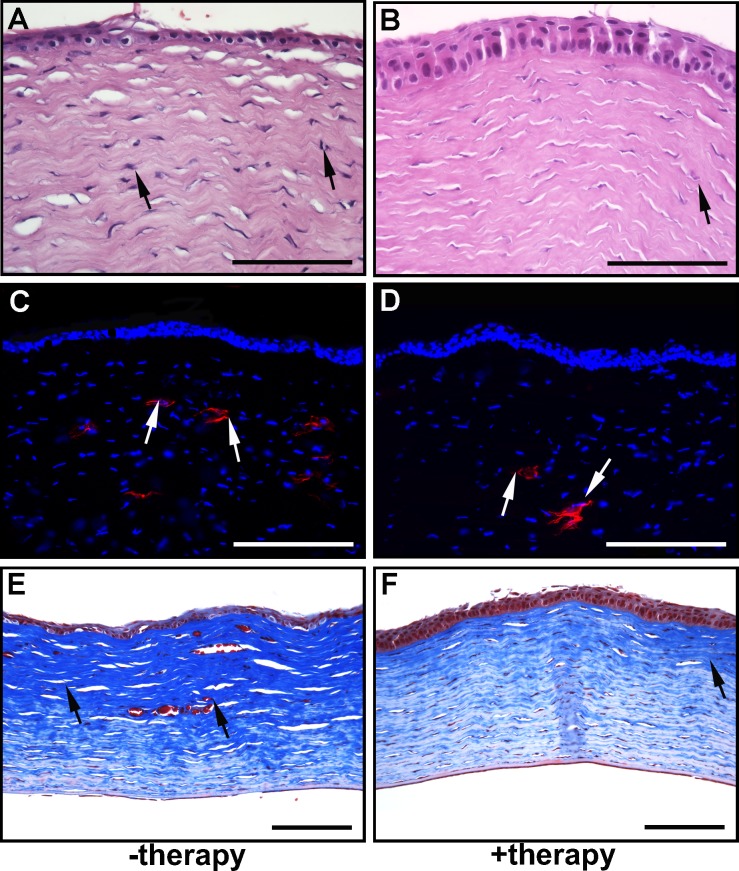
Combination BMP7 and HGF gene therapy is safe and caused no adverse effects in rabbit eyes. H&E images show no alteration in corneal morphology with PEI2-GNPs alone (A) and with BMP7+HGF gene therapy (B). CD11B immunofluorescence images 21 days after corneal injury show no infiltration of leukocytes and inflammatory markers in the stromal layer in corneal tissue from PEI2-GNP–treated rabbits (−therapy) and from BMP7+HGF-treated rabbits (+therapy). Masson's trichome–stained corneal tissue images show decreased collagen expression in BMP7+HGF-treated eyes (F) compared to the −therapy given eyes (E). Scale bar: 100 and 200 μm.

H&E staining demonstrated noteworthy morphologic alterations in corneal epithelium and stroma at day 21 post alkali injury in the rabbits that received PEI2-GNP-naked vector (Fig. 10A), corroborating the presence of the clinical opacification visualized with slit-lamp biomicroscopy. *BMP7*+*HGF* therapy markedly mitigated the adverse impact of alkali wounding on corneal epithelial and stromal tissues (Fig. 10B). In addition, no significant cellular inflammatory infiltrates were observed in the corneas of either group, as quantified with CD11b immunofluorescence (Figs. 10C, 10D; *P* > 0.1). The Masson's trichome staining demonstrated notably decreased collagen deposition in the rabbit corneas that received *BMP7*+*HGF* therapy (Fig. 10F) compared to the −therapy naked vector–delivered corneas (Fig. 10E).

### Role of *HGF* in Apoptosis of Corneal Myofibroblasts and Fibroblasts

**Figure 11:**
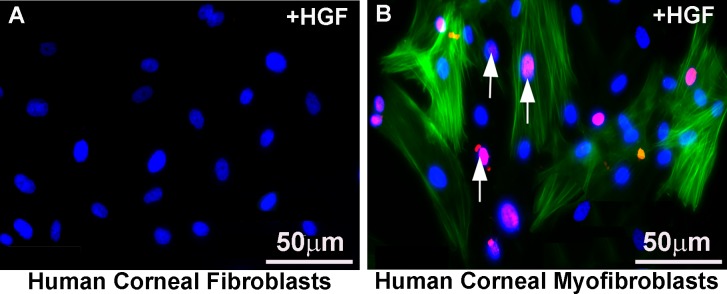
Immunocytochemistry images of α-SMA staining (green) and TUNEL staining (pink) in cell cultures of HCFs (A) and myofibroblasts (B) after rHGF treatment, demonstrating that rHGF selectively induces apoptosis of corneal myofibroblasts. Arrows point to TUNEL-positive myofibroblast cells. Scale bar: 50 μm.

The molecular function of *HGF* on corneal fibroblasts and myofibroblasts was studied in an established in vitro model of corneal fibrosis in humans. Primary stromal cultures obtained from donor human corneas and grown in the absence of TGF-β1 provided corneal fibroblasts and grown in the presence of TGF-β1 produced corneal myofibroblasts, as demonstrated by *α-SMA* staining and TUNEL staining. Recombinant *HGF* (r*HGF*) treatment of these cultures caused significant apoptosis, as indicated by the detection of several TUNEL-positive cells in human corneal myofibroblasts (Fig. 11B) but not in HCFs (Fig. 11A).

**Figure 12:**
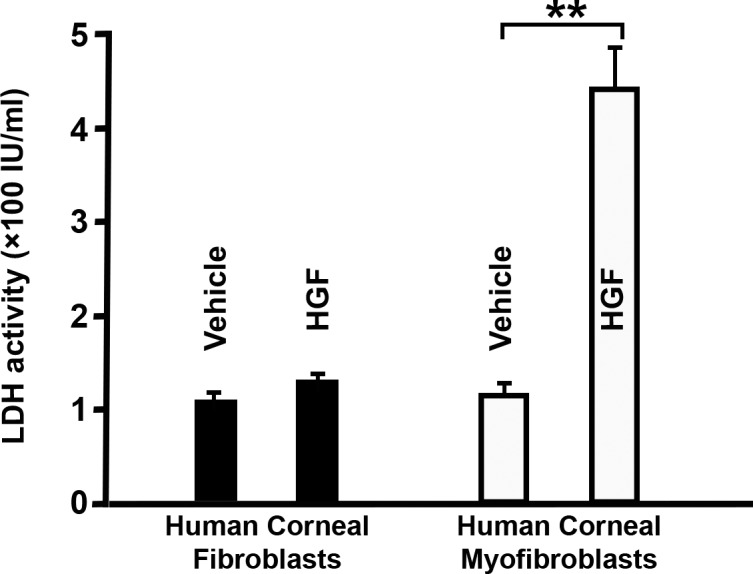
Elevated LDH activity in cells treated with rHGF. Graph depicting the levels of LDH measured in HCFs and myofibroblasts treated with vehicle or rHGF. Error bars represent ± SEM (*P < 0.001 against vehicle control in respective group).

These observations were further validated by the LDH assay, which measures cell death by gauging LDH enzyme released by apoptotic cells. As Figure 12 shows, LDH values were significantly higher in r*HGF*-treated corneal myofibroblasts than in vehicle-treated myofibroblasts (442 ± 40 vs. 130 ± 11 IU/mL; *P* < 0.001). With corneal fibroblasts, LDH values did not show marked differences between r*HGF*-treated and the vehicle-treated cells (123 ± 8 vs. 136 ± 9 IU/mL).

## Discussion

The pathogenesis of corneal haze from mechanical, chemical, or surgical injury is a complex cascade of molecular signaling events that involve a variety of fibrotic pathways and ECM remodeling factors.^3,4^ These events are primarily controlled by the TGF-β signaling pathway.^34,46^ Corneal wound healing involves differentiation of dormant keratocytes to active fibroblasts and myofibroblasts, which proliferate, secrete ECM, and aid in wound contraction and closure.^4,47,48^ Continued activation of myofibroblasts in the cornea, however, leads to aberrant collagen deposition and corneal haze and fibrosis,^3,47^ making regulation of myofibroblast activation an important therapeutic target for controlling and preventing corneal scarring.

TGF-β signaling is regulated in numerous pathways and has a variety of functions in tissue homeostasis. It also negatively regulates the BMP pathways,^49^ making it a challenge to directly regulate the TGF-β pathway. *BMP7* negatively regulates the TGF-β signaling processes in corneal tissue remodeling by counteracting the epithelial mesenchymal transition pathways, which contribute to an increase in fibrosis.^17,50^ We previously demonstrated in a rabbit model the efficacy of GNP-mediated *BMP7* gene transfer in reducing corneal opacity following injury.^17^ *HGF* is another important regulator of the wound-healing process in various tissue types^51^ and is reported to function through c-Met and β1-integrin signaling pathways.^23^ Our research team and others have demonstrated that *HGF* plays a role in corneal wound healing,^22^ but to our knowledge its efficacy in promoting in vivo wound healing has never been studied in the eye, although such an effect has been described in models of lung,^39^ kidney,^37^ hepatic,^52,53^ and skin wound healing.^51^

We hypothesized that *HGF* gene transfer in combination with *BMP7* locally into the opaque cornea could reverse the fibrotic events in vivo in a preclinical rabbit model of corneal fibrosis. The results of this study demonstrate that delivery of *BMP7*+*HGF* genes into stromal fibroblasts/keratocytes via nanoparticle (PEI2-GNP) significantly reduced corneal opacity by 3 weeks post injury in a preclinical rabbit model of corneal fibrosis (Figs. 1–4). Importantly, since the *BMP7*+*HGF* therapy was administered 1 day after injury, the findings suggest that resolution of corneal opacity and vision restoration is achievable even in a significantly damaged cornea in vivo. Furthermore, administration of *BMP7*+*HGF* gene therapy was associated with a significant reduction in *α-SMA*, a molecular marker for myofibroblasts, and a concomitant decrease in profibrotic genes (Figs. 5 and 6). To corroborate the selective apoptosis observed in rabbit myofibroblasts was driven by *HGF*, we performed in vitro studies of the effects of r*HGF* on human corneal myofibroblasts and fibroblasts, which demonstrated that r*HGF* induces apoptosis in human corneal myofibroblasts but not in HCFs (Figs. 11 and 12). This finding aligns with earlier reports of *HGF* function in pulmonary and liver fibrosis models, which suggests that *HGF*-induced myofibroblast cell death is a key event in the healing process.^53^ TGF-β signaling has been shown to increase the expression of c-Met, the receptor for *HGF*,^54^ suggesting that in a fibrotic microenvironment the *HGF* activity may be more pronounced in myofibroblasts than in quiescent stromal keratinocytes. The c-Met receptor, a proto-oncogene, has multiple functions, including cell survival via sequestration of the Fas receptor and inhibition of death-domain–induced signaling.^55^ However, in presence of *HGF*, the binding of c-Met with Fas receptors would be dampened due to c-Met activation by phosphorylation, and the cells can then respond to Fas ligand–dependent death signals.^56^ In addition, *HGF* also induces increased caspase-3 activity via intracellular signaling.^57^ Caspase-3–dependent cleavage of the c-Met receptor converts it into a 40-kDa proapoptotic signal.^58^ Therefore, the selective death of myofibroblasts observed by *HGF* treatment could be the result of a combination of three factors: increased c-Met receptors on myofibroblasts, elevated caspase activity, and the generation of proapoptotic c-Met fragments. *HGF* has also been shown in lung fibrosis models to increase matrix metalloproteinase expression, leading to cell death and a concomitant reduction in ECM.^39^ Hence, increased matrix metalloproteinase levels could be a mechanism to prevent corneal scar formation and aberrant rearrangement of the ECM in the presence of *HGF*.

On the other hand, *BMP7* is well known to antagonize the TGF-β pathway^15,17^ via upregulation of the Id3 proteins^59^ and Smad 7 signaling.^60^ Coadministration of *BMP7* and *HGF* has been found therapeutic in renal fibrosis and tubular nephropathy.^61^ Expression of profibrotic factors such as *α-SMA*, fibronectin, and collagens has been shown to decrease in response to *BMP7* treatment.^17^ Therefore, the two therapeutic genes, *BMP7* and *HGF*, represent two independent pathways for controlling corneal wound healing without scarring, namely by regulating TGF-β signaling and by selective apoptosis of myofibroblasts. Such a combination treatment modality, targeting the initial signaling phase (via *BMP7*) and the downstream myofibroblast apoptosis and resolution (via *HGF*), can therefore have a greater potential for disease resolution even in advanced cases of fibrosis. To the best of our knowledge, this is a first study that demonstrates elimination of established corneal scar and restoration of corneal transparency in vivo by activating multiple signaling pathways. This innovative approach is expected to lead development of the first nonsurgical gene-based therapies to cure preexisting corneal fibrosis/scarring without significant adverse effects.

Data from decades of clinical trials in other fibrotic diseases,^62^ including renal^63^ and pulmonary fibrosis,^64^ suggest that single-targeted therapy is typically less effective in disease resolution. In the clinical setting, patients who have incurred a corneal injury generally have well-established corneal haze (fibrosis) at the time of presentation to an ophthalmologist. An efficient gene delivery method as well as a multimodal-targeted treatment approach may offer the potential for achieving better results than what is possible with currently available therapies. Our study found that PEI2-GNP–delivered *BMP7*+*HGF* genes remained in the injured rabbit cornea at high copy numbers for up to 3 weeks after instillation (Fig. 8) without concomitant adverse ocular affects such as an increase in IOP (Fig. 9) or dry eye conditions. Furthermore, Draize and modified MacDonald-Shadduck scores (Table) indicate that ocular toxicity from *BMP7*+*HGF* combination gene therapy is minimal. Nevertheless, the long-term effects of *HGF*+*BMP7* therapy on stromal collagen fibril organization and neighboring ocular tissues remain unknown and warrant further investigation. The lack of data on long-term effects is a limitation of the present study, and our future studies will investigate the long-term effects of PEI2-GNP–delivered *BMP7*+*HGF* gene therapy. In conclusion, this study demonstrates that tissue-targeted *BMP7*+*HGF* combination gene therapy given locally with PEI2-GNPs 1 day after alkali damage eliminates corneal fibrosis and restores corneal transparency in vivo by negating TGF-β pathologic activity producing myofibroblasts and promoting apoptosis in established myofibroblasts utilizing multiple signaling pathways.
